# Establishment of a Rapid Phone Consultation Service for COVID-19 Public Health Support in Nepal

**DOI:** 10.7759/cureus.27977

**Published:** 2022-08-13

**Authors:** Ishani Singh, Navindra R Bista, Kabin Maleku, Prabhat Adhikari, Anu Hyanmikha, Santosh Pradhan, Ramu Kharel

**Affiliations:** 1 Medicine, Kathmandu Medical College, Kathmandu, NPL; 2 Anesthesiology, Tribhuvan University Institute of Medicine, Kathmandu, NPL; 3 Telemedicine, ASK Foundation, Kathmandu, NPL; 4 Infectious Diseases and Critical Care, Danphe Care, Kathmandu, NPL; 5 Infectious Disease and Critical Care Specialist, Center for American Medical Specialists, Kathmandu, NPL; 6 Hospital Administration, Tata Institute of Social Sciences, Kathmandu, NPL; 7 Information Technology, Kosi Collaborative, Kathmandu, NPL; 8 Emergency Medicine, Brown University, Providence, USA

**Keywords:** covid-19, lalitpur, kathmandu, hotline, telecommunication, vpn, sip line, telemedicine, nepal, pandemic

## Abstract

Background

The coronavirus disease 2019 (COVID-19) pandemic strained the already weak health system of Nepal, especially during the surge of the delta variant. A telephonic consultation service was rapidly established to provide free consultations to assist those in home isolation due to severe acute respiratory syndrome coronavirus 2 infection. In this study, we describe the process of establishing the hotline and share preliminary findings. During the peak of the delta wave in Nepal, the hotline was started by a local non-profit organization.

Methodology

We established the hotline with help of a private telecommunication company. The hotline was advertised on social media, radio, and newspapers. Healthcare workers were recruited and trained and the service was provided for free. Patient data were recorded and de-identified for analysis, monitoring, and evaluation.

Results

The majority of the callers were from Kathmandu valley, which includes three districts, Kathmandu, Lalitpur, and Bhaktapur. Overall, 44% of the callers inquired about the clinical manifestations of COVID-19. On average, there were 75 calls each day between May 2021 and February 2022. The average call duration was three minutes and 42 seconds. Trained healthcare workers answered the calls for 15.5 hours a day.

Conclusions

Our work established the feasibility of a rapid hotline service in response to the pandemic causing high strain on the health system. Lessons learned from this experience can be useful for future disasters in Nepal and other places with similar health system strains.

## Introduction

The coronavirus disease 2019 (COVID-19) pandemic pushed health systems to extreme limits worldwide, especially in Nepal where the pre-existing health infrastructure is considered to be one of the weakest in the world [1]. Since Nepal’s first case was reported on January 23, 2020, COVID-19 spread in the community across the country with nearly 978,155 cases and 11,950 deaths by mid-February 2022 [2]. While the Nepal government had a mitigation plan to fight the spread of the virus, the country’s health infrastructure collapsed during the delta wave of the pandemic [3]. In the health sector emergency response plan, the Nepal government proposed to provide health services for both COVID-19 and non-COVID-19 patients, as well as mass counseling through telemedicine by contacting listed doctors via SMS, phone, email, and other means of communication. While the country had some of the highest per-capita cases of COVID-19 in the world, patients were also dying due to a lack of basic resources, including oxygen, essential medications, hospital beds, and transport systems [4]. Nearly 80% of the COVID-19 cases were mild and could be managed at home [5]. In the midst of COVID-19, the healthcare system was overburdened with people who could have been cared for at home.

Telemedicine has been around since 1879 and has grown significantly with technological advancements [6]. During the COVID-19 pandemic, countries outside of Nepal established telemedicine services to support the infected public such as Sesame Care, PlushCare, Teladoc, MeMD, HealthTap, Amwell, MDLive, and Doctor on Demand in various parts of the world [7]. Nepal’s health system is severely constrained by geography, a lack of access to healthcare services, and poor infrastructure. For a population of nearly 30 million, Nepal was reported to have 9.6 critical care beds per 100,000 people (2,797 intensive care unit (ICU) beds as per the 2021 census), 1,008 ventilators, and only 16,432 physicians before the start of the delta wave [3,4]. In Nepal, there are only 67 doctors and nurses per 100,000 population compared to the World Health Organization (WHO) recommendation of 230 health professionals for every 100,000 people [5]. A phone hotline or telemedicine has proven very effective in countries with poor health systems [8].

To help with the rising burden on the pre-existing health system of Nepal, ASK Foundation, a non-profit organization based in Nepal, started a free phone consultation service for those infected with COVID-19. ASK Foundation has worked in the field of telemedicine service across Nepal for a decade and was able to strengthen the previously established centers to execute the COVID-19 hotline rapidly. The foundation started general telemedicine services, initially as HTP Telehealth Innovation Foundation, and later got registered as ASK Foundation in 2016. The service was started in remote parts of Nepal in 2009 with the help of four medical graduates, a philanthropist, and an engineer who collaborated with the rural welfare council. ASK foundation has successfully provided telemedicine services for 8,000 patients free of cost in a span of five years. The foundation is making every effort to overcome obstacles and limitations to keep delivering telemedicine to current centers and to extend services to additional centers across the nation during the ongoing rising COVID-19 cases.

This paper describes the process of creating the hotline and preliminary findings from 10 months of free COVID-19 hotline phone service across Nepal.

## Materials and methods

Establishing the hotline

ASK Foundation has been working in telemedicine for a long time in collaboration with local government agencies and with the approval of the social welfare council. The hotline is an extension of the service to the general public.

Obtaining and setting up the phone line

The telecommunication line and an easy-to-remember phone number were provided by a private telecommunication company, registered under Nepal Telecommunication Authority. To lower the risk of COVID-19 transmission among healthcare workers, a hotline service was set up from the home of the healthcare worker. A SIP line (session initiation protocol) was implemented to ensure a phone line capable of making multiple calls (up to 10 incoming or outgoing). The get desk software handled all calls. Through a VPN (virtual private network), the software was installed on each healthcare worker’s personal computer. All healthcare professionals installed social media platforms, in addition to the hotline telecommunication system, for communication with consultants and patients if expert support was required.

Recruitment and training of healthcare workers

Consultant physicians (one) (MDs), medical officers (two) (MBBS), and nurses (four) were recruited to answer the incoming phone calls. Vacancy announcement was made through social media platforms, and candidates were hired by ASK Foundation. They were trained on a wide range of topics, including community-level clinical management of COVID-19, infection prevention and control (IPC), phone call operational management, and communication strategies. Each training is described in detail below.

Clinical Management Training

The healthcare workers participated and got certified by Brown University and Project HOPE’s training on clinical management of COVID-19 [9]. The topics of the three hours of training included an introduction to COVID-19, common signs and symptoms of the disease, as per the Centers for Disease Control and Prevention (CDC) guidelines for COVID-19 management and National Medical Commission (NMC) guideline for disease categorization, care escalation in COVID-19, and IPC measures. Further training on prescription writing format and nutrition consultation during COVID-19 was also provided. All training was provided by physicians and public health professionals directly working with COVID-19 patients.

Communication Training

A two-hour communication training was given to providers by physicians and communication consultants and focused on patient-centric communication styles. All healthcare workers ran through multiple case scenarios. Patient communication support in difficult cases where symptoms were more severe or when questions arose that could not be answered by the phone providers, and on-call MD consultants were available to support.

Operational Training

Training on the get desk software, use of electronic medical records, and patient privacy was also conducted. A pre-operational mock drill was also conducted to ensure the appropriate use of the software and forms.

Dissemination/Advertisement of the hotline number

After the establishment of the hotline, advertisement of the hotline was done through various news media, social media, and organizations that were actively working on COVID-19 management, local government bodies, and large organization networks. Partnerships with radio stations and social media influencers were formed to disseminate the hotline information rapidly.

Electronic record and data management

Get desk software was used to collect data on call distribution patterns and several calls responded by the healthcare worker. Patient data were recorded and de-identified for analysis, monitoring, and evaluation. The day-to-day activities were summarized and reflected and visualized in the Google Data Studio.

Funding

The setup cost of establishing the SIP, outgoing calls, and healthcare worker’s training line was nearly 40,000 NPR. Personnel costs included healthcare workers’ salaries and ancillary staff. Health care workers were paid 30,000 NPR ($235) for the doctors and 18,000 NPR ($146) for the nurses. The cost was covered by ASK Foundation through its wide range of donor organizations.

## Results

The phone service was active from May 1, 2021, and continued through the omicron wave. The service was provided for 15.5 hours a day (6 am to 9:30 pm). The time was chosen after reviewing call volume distribution to cover most of the calls. In case there were calls during the 9:30 pm-6 am time period, providers called back the next day.

Call distribution

**Figure 1:**
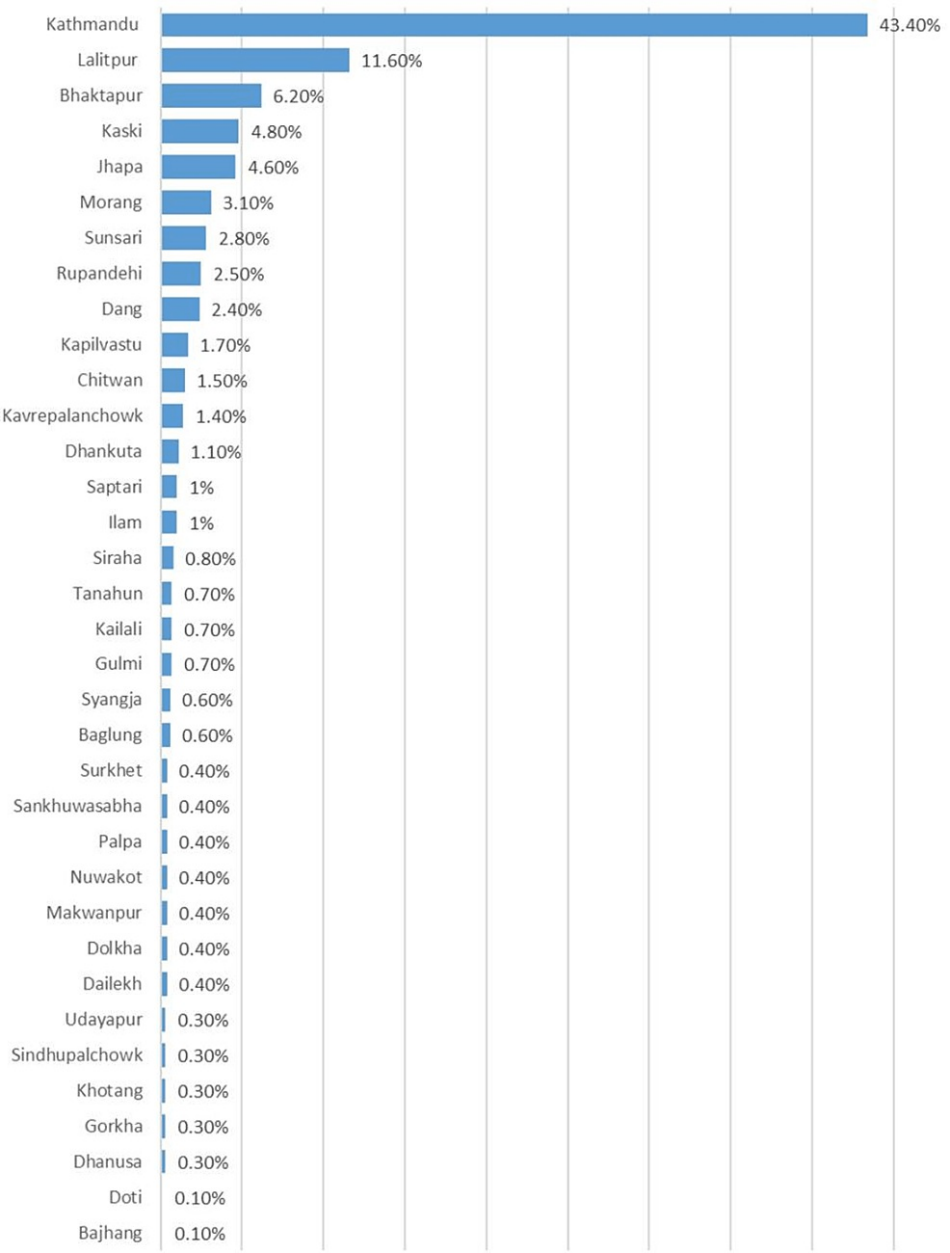
Geographical call distribution.

The total number of incoming calls was 12,555. In total, 821 calls were missed, and 1,525 calls were outbound for follow-up purposes (Table 1). The majority of the callers were from the Kathmandu valley, which includes three districts, Kathmandu, Lalitpur, and Bhaktapur (Figure 1). As the region with the highest burden of COVID-19 in the country, we expected more calls from the valley. According to the Nepal government data, 43% of total cases till February 2022 have been from the Kathmandu valley [10]. Accessibility to the internet or limited phone service in the rural areas of Nepal could be a potential cause. Furthermore, advertisement of the hotline and advocacy programs conducted by the ASK Foundation during the peak of the delta wave was centered around the Kathmandu Valley. Besides Kathmandu Valley, Kaski, Bhaktapur, Lalitpur, and Rupandedhi were the districts with higher participation.

**Table 1:** Total number of calls from May 2021 till February 2022.

Incoming calls	Missed calls	Outbound calls
12,555	821	1,525

Call timings

**Figure 2:**
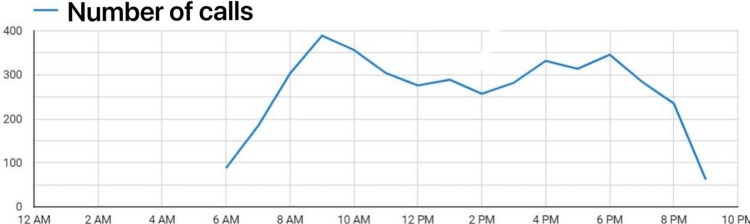
Average call volume time during a day between May 2021 till February 2022.

Most calls were received in the morning and evening times, as depicted in Figure 2. During the peak of the delta wave, 329 calls were received on the highest call volume day. On average, there were 75 calls each day in the months of May and June 2021. The average call duration was three minutes and 42 seconds. The majority of the callers were males (58.6%).

Call queries

**Figure 3:**
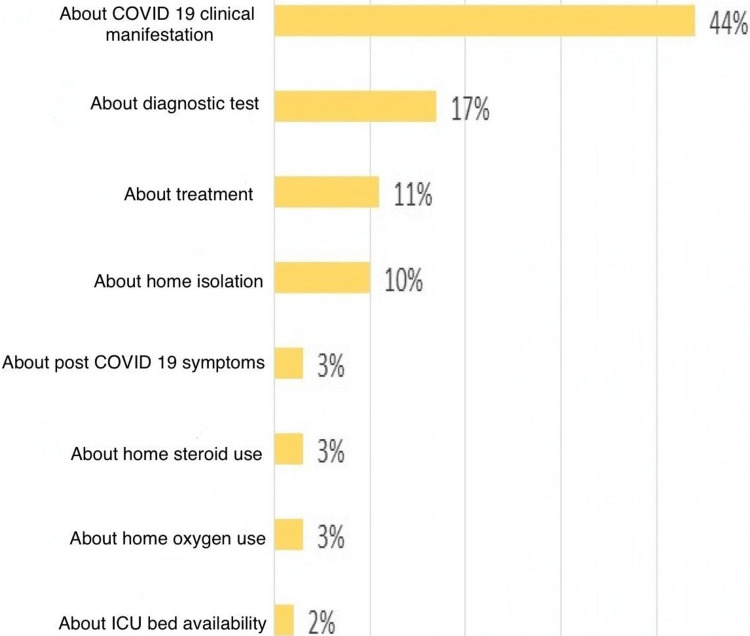
Categories of queries asked in COVID-19 hotline service. COVID-19: coronavirus disease 2019

Overall, 44% of the callers inquired about the clinical manifestations of COVID-19, whereas others called to inquire about diagnostic tests, treatment methods, and home isolation-related queries. Other questions included symptomatic management and critical signs/symptoms related to COVID-19 that could cause potential clinical deterioration. While the majority of the calls were about their personal health and other problems (which included food support, financial support in COVID-19, etc.), others had called on behalf of their relatives and friends as well. Figure 3 shows the category of queries that were asked. 

## Discussion

In our experience, this rapid service was widely used during the peak of the pandemic, with queries regarding COVID-19 comprising the majority of calls. Our service provided home-care suggestions while also providing information on logistics such as nearby ambulances, hospitals, oxygen cylinder resources, and local necessary phone numbers. For calls that were missed during the off-hours, an active effort was put to return the calls on the following day. Our team tagged certain calls for follow-up based on the severity of the case and the need for extra information.

The rapid hotline was possible owing to a multidisciplinary team working together that included doctors, nurses, pharmacists, and information technology experts. We designed our shift schedule to cover the maximum number of patients possible during peak times. Prescription and medication suggestions were given through social media messengers and pre-existing ASK Foundation telemedicine infrastructure.

Our hotline service during COVID-19 provided free consultation with trained individuals providing the service throughout the day and the duration of the high burden period in Nepal. Our experience provides a blueprint for organizations to set up rapid hotlines in times of acute disasters in the future. Additionally, we discovered that individuals needed assistance during outbreaks in addition to concerns about the disease itself. People isolated due to infectious disease may experience negative psychological reactions such as post-traumatic stress, anxiety, anger, and reinfection which need proper addressing [11]. Although telemedicine might not solve all these concerns at once, it can provide easier access to care for their psychological concerns [12]. Telemedicine has been utilized and recognized in various settings, including unfortunate climates and critical fiascos; moreover, the utilization of telemedicine innovation in calamity and crisis is not as normal or as strong as we would want. As in this pandemic, reactions to disasters might include different nations; however, with regards to telemedicine, the viability of these is not entirely settled by a few factors, including the specialized foundation, modalities, and human limits present before the beginning of the disaster [13,14].

It may be less expensive to schedule a telephone consultation than to schedule one in person. The only controlled trial of daytime telephone triage by general physicians revealed that patients handled in this manner were 50% more likely to re-consult within two weeks than those seen in person [15]. Our experience shows that a hotline number such as ours can help lower the burden on the health system during an acute public health stressor event. This is evident by the volume of calls and queries we received on this phone line. We also received feedback and patients had a very positive response regarding our service. In fact, our service was well received and we still have callers referencing our name. Our number is still being circulated and it is all by word of mouth. Furthermore, in a country with poor health infrastructure, telemedicine and hotline services can pave the way to increase accessibility in the future.

Limitations

Though this work was successful based on the number of calls answered and the utilization of the phone service, key limitations were noted as well. First, while the service was provided for free, there was a cost for the user to make the phone call. Setting up a toll-free number would have likely made the service more accessible to the needed population.

Likewise, the provided number was relatively easy, but it was a nine-digit number. Having a shorter (three-digit) number could have improved the number of incoming calls. Medical consultation was provided using audio, and this had its challenges, especially in objectively evaluating a patient’s condition. For example, it would have been easier to assess the severity of a COVID-19 patient if we could see via a video call, the way they were breathing, instead of them just describing it on the phone. Future iterations could include video components that would help the healthcare professional to build a better rapport with the patient.

Further, this service was only available during day time. Though our team called back the phone calls that were missed, the accessibility of services could have been expanded to 24 hours. If we had received additional financial assistance, we would have increased our service hours by recruiting more healthcare professionals. We sought help from the Government of Nepal as well but they were not in a position for any financial help at the time.

Our calls were also seen mostly from the Kathmandu valley, and this is likely due to the highest burden of the cases being from the Kathmandu valley, as well as due to the easy accessibility of technology in the city. The language was likely another barrier to the expansion of the service across Nepal as the services were advertised in English, which is not the language spoken by the majority. Rural regions of Nepal, especially the ones bordering India, had a high number of deaths during the delta wave. These regions must be focused on such services by provision of a widely used telecommunication system.

## Conclusions

Telemedicine during the COVID-19 pandemic was a very efficient and safe way to reach out to healthcare professionals by the public. It is expected that telemedicine will help reduce the burden on hospitals and will help triage and treat or refer the patient according to their need. In developing countries like Nepal, telemedicine is yet to be an integral part of the healthcare system, which, if utilized properly, can increase access to healthcare. Telephonic consultation during the COVID-19 pandemic was found to have high utility, and this work paves the path for the future use of such technologies in Nepal.
